# Chronic Obstructive Pulmonary Disease in Cameroon: Prevalence and Predictors—A Multisetting Community-Based Study

**DOI:** 10.1155/2023/1631802

**Published:** 2023-09-13

**Authors:** Massongo Massongo, Adamou Dodo Balkissou, Laurent-Mireille Endale Mangamba, Virginie Poka Mayap, Marie Elisabeth Ngah Komo, Abdou Wouoliyou Nsounfon, Alain Kuaban, Eric Walter Pefura Yone

**Affiliations:** ^1^Faculty of Medicine and Biomedical Sciences, University of Yaoundé I, Yaoundé, Cameroon; ^2^Faculty of Medicine and Biomedical Sciences, University of Ngaoundéré, Garoua, Cameroon; ^3^Faculty of Medicine and Pharmaceutical Sciences, University of Douala, Douala, Cameroon; ^4^Laquintinie Hospital, Douala, Cameroon; ^5^Jamot Hospital, Yaoundé, Cameroon; ^6^Central Hospital of Yaoundé, Yaoundé, Cameroon

## Abstract

**Objective:**

Little is known concerning chronic obstructive pulmonary disease (COPD) in Sub-Saharan Africa (SSA), where the disease remains underdiagnosed. We aimed to estimate its prevalence in Cameroon and look for its predictors.

**Methods:**

Adults aged 19 years and older were randomly selected in 4 regions of Cameroon to participate in a cross-sectional community-based study. Data were collected in the participant's home or place of work. Spirometry was performed on selected participants. COPD was defined as the postbronchodilator forced expiratory volume in 1 second/forced vital capacity ratio (FEV1/FVC) < lower limit of normal, using the global lung initiative (GLI) equations for Black people. Binomial logistic regression was used to seek COPD-associated factors. The strength of the association was measured using the adjusted odds ratio (aOR).

**Results:**

A total of 5055 participants (median age (25^th^-75^th^ percentile) = 43 (30–56) years, 54.9% of women) were enrolled. COPD prevalence (95% confidence interval (95% CI)) was 2.9% (2.4, 3.3)%. Independent predictors of COPD (aOR (95% CI)) were a high educational level (4.7 (2.0, 11.1)), living in semiurban or rural locality (1.7 (1.4, 3.0)), tobacco smoking (1.7 (1.1, 2.5)), biomass fuel exposure (1.9 (1.1, 3.3)), experience of dyspnea (2.2 (1.4, 3.5)), history of tuberculosis (3.6 (1.9, 6.7)), and history of asthma (6.3 (3.4, 11.6)). Obesity was protective factor (aOR (95%CI) = 0.3 (0.2, 0.5)).

**Conclusion:**

The prevalence of COPD was relatively low. Alternative risk factors such as biomass fuel exposure, history of tuberculosis, and asthma were confirmed as predictors.

## 1. Introduction

Chronic obstructive pulmonary disease (COPD) is characterized by airflow limitation causing respiratory symptoms such as cough and shortness of breath, acute episodes known as exacerbations, and a progressive decline of lung function, which can lead to chronic respiratory failure and disability, with a great impact on quality of life. This is a growing and threatening public health problem worldwide, with an estimated 251 million cases in 2016 [1]. The number of COPD cases has been increasing in the past decades. According to Adeyole et al.'s systematic review relying on 123 studies and more than 877, 000 participants, the estimated COPD cases increased by 68.9% worldwide and 102% in Africa between 1990 and 2010 [2]. Spirometry-diagnosed COPD prevalence varies widely from one country or region to another, and even within countries and regions, ranging from less than 3% to more than 35% [3–5]. This prevalence appears to be low in the poorest regions of the world and to rise when the mean number of pack-years smoked increases [4].

Mortality associated with COPD is also increasing, and projections put it as the 4^th^ leading cause of death worldwide as well as in low-income countries (LIC) by 2030 [6]. Up to 90% of those deaths currently occur in low- and middle-income countries (LMIC) [1]. The most commonly cited cause of COPD is exposure to tobacco smoke. Other well-known risk factors for COPD include advancing age [5, 7, 8], occupational and outdoor exposures [8–11], and low birth weight or childhood respiratory disorders [8]. However, evidence is growing on alternative risk factors such as biomass fuel [7, 11–14], history of tuberculosis (TB) [15–22], or socioeconomic status [5, 7, 23], especially in LMIC. Much more, over 3 billion people are exposed to biomass fumes, while only 1 billion are exposed to tobacco smoking [12].

In Sub-Saharan Africa, mostly made up of LIC, there has been a marked increase in COPD prevalence and mortality; studies, however, had been relatively scarce prior to 2010. Their numbers increased during the past decade, although most of them originated from Eastern [24–26] and Southern [13, 27–31] Africa. We found only 3 recent COPD-related studies originating from West or Central Africa: 2 of these were hospital-based and human immunodeficiency syndrome (HIV) related [32, 33], and only one was community-based, which is part of the present study [34]. Much more, COPD-related awareness is poor among health workers as well as the community [13, 27]. We aimed to assess COPD prevalence and look for its determinants among an adult Cameroonian population.

## 2. Materials and Methods

### 2.1. Design, Setting, and Period

This was a community-based cross-sectional study, led from 2014 to 2018 in 5 localities (2 urban, 2 semiurban, and 1 rural) in Cameroon, a central African country with an estimated 23794164 inhabitants in 2018 [35], who are divided into 4 cultural groups: Fang-Beti, Grass fields, Sawa, and Sudano-Sahelian. Participants were recruited during 4 distinct periods of 5-6 months each: from December 2013 to May 2014 in Yaoundé, from December 2015 to April 2016 in Bandjoun, from December 2016 to May 2017 in Douala, and from December 2017 to April 2018 in Garoua and Figuil. Yaounde is the nation's capital, located in the Centre Region, with a cosmopolitan but mainly Fang-Beti population that was estimated at 1817524 in 2018. Douala is the country's economic capital, with a population of 1907479 inhabitants, who are also cosmopolitan and primarily of the Sawa cultural group. Yaounde and Douala were 2 urban settings. Bandjoun (West Region, 65 021 inhabitants) and Garoua (North Region, 265 583 inhabitants) were the 2 semiurban settings, with Grass fields and Sudano-Sahelian as the main cultural groups, respectively. Figuil (North Region, 67 997 inhabitants) was the only rural setting with a mainly Sudano-Sahelian population. Each of these localities was divided into count zones used in the 2005 national census [35] and included various numbers of health areas defined by the national health system.

### 2.2. Population and Sampling

Adults aged 19 years and above, living in the study area, and free from cognitive, auditory, or language impairments, were randomly invited to participate following a 3-level clustered sampling. At the first level, given numbers of count zones (CZ) and health areas (HA) were randomly selected in urban and semiurban/rural settings, respectively. At the second level, households were selected using a systematic random sampling, with a rate of 1 over 2-3 in CZ and 1 over 1-14 in HA. At the third level, all people aged ≥ 19 years in the selected households were invited to participate in the study. Those who declined our invitation and those who did not complete the study questionnaire were excluded. Given the limited time and device availability, a spirometry was randomly proposed to half of the included participants.

We calculated our required sample size with the StatCalc tool of Epi Info version 7.2.3.1 (EPI INFO™, Center for Disease Control and Prevention, USA), using a dedicated formula for population surveys. We considered a 95% confidence interval, 5% expected prevalence found by Pefura-Yone et al. in Yaounde and Foumbot (Cameroon) a year before this study [32], a 1% margin of error, a design effect of 2, and a 10% nonresponse rate. The obtained sample size was 4501.

### 2.3. Data Collection and Variables

Seventh year trained medical students collected data, during a face-to-face interview using an electronic questionnaire. The anamnestic data included demographic (gender, age, and residency) and socioeconomic (level of education, marital status, ethnicity, and socioeconomic level) data, tobacco smoking and alcohol consumption history, biomass fuel exposure, and medical history (tuberculosis, asthma, pneumonia, high blood pressure, heart disease, and diabetes). Respiratory symptoms (chronic cough, chronic expectoration, and dyspnea), anthropometric parameters (weight, height, body mass index, and related categories), and spirometry data (forced expiratory volume in 1 second (FEV1), forced vital capacity (FVC), and FEV1 over FVC ratio (FEV1/FVC), as well as their lower limits of normal (LLN)) were also collected.

Anamnestic data were collected by questioning the subject or a relative. Demographic data, socioeconomic data, habits, and symptoms were self-reported. Data on medical history were those reported as diagnosed by a health professional. The height was measured by a wooden measuring board of local manufacture graduated in centimeters, and the weight by an electronic weighing scale. The body mass index (BMI) was calculated as weight (in kg)/height^2^ (in m^2^) and used to divide the study population into 4 weight categories: lean (BMI < 18), normal (BMI ≥ 18 and <25), overweight (BMI ≥ 25 and <30), and obese (BMI ≥ 30). Biomass fuel exposure was defined by the use for more than 6 months of either coal, charcoal, wood (logs or chips), sawdust, dung, or crop residues for heating or cooking purposes. Tobacco smoking was categorized as “never smoked” (less than 20 cigarettes for the whole lifespan) and “ever smoked” which was subsequently quantified in pack-years.

Spirometry measurements were performed using a digital turbine pneumotachograph (Spiro USB, Care fusion, Yorba Linda-USA), following American Thoracic Society standards and European Respiratory Society guidelines [36, 37]. Each selected participant was comfortably seated and was asked to perform the procedures after suitable explanations. Prebronchodilator procedures were first performed, measuring FEV1, FVC, and FEV1/FVC. Those with FEV1/FVC < 0, 70, defined as airflow limitation (AFL), were asked to inhale 400 *μ*g of pressurized salbutamol before undergoing a postbronchodilator procedure 20 minutes later, to check the reversibility of obstruction. Those with no AFL were considered to not have any ventilator obstructive defect. The LLNs for postbronchodilator FEV1/FVC were determined using the global lung initiative (GLI) 2012 Data Conversion tool (Global Lung Initiative Version 1.3.2, ©2012 PH Quanjer, S Tanojevic, TJ Colze, J Stocks) for Black people.

COPD was defined as a FEV1/FVC < GLI − defined LLN. Prebronchodilator FEV1 was used to classify COPD in severity stages, according to the first Global Obstructive Lung Disease (GOLD) classification [38].

### 2.4. Data Management and Analysis

The collected data were recorded directly in the Epidata entry 3.1 software. Analyses were done using R, Version 4.0.2. Qualitative data were presented as counts (proportions) and quantitative variables as medians (25th-75th percentiles).

The association between COPD and independent variables was tested by using binary logistic regression and determined by the odds ratios (OR) and their 95% confidence interval (95% CI). A univariate analysis was first performed with the crude OR (cOR). Variables associated with COPD with a *p* value < 0.10 were eligible for a step-down sequential multiple logistic regression to determine the adjusted OR (aOR). The latter stopped when any variable with a nonsignificant association (*p* value ≥ 0.05) had been removed, unless it had been identified as a confounder (removal causing model destabilization or a >30% change in aOR of another variable).

## 3. Results

### 3.1. Population Characteristics

**Figure 1:**
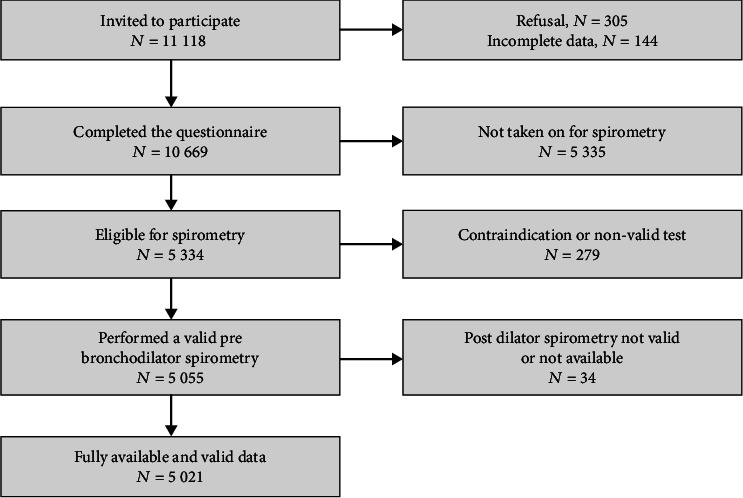
Flowchart of participant's enrollment in COPD community study, Cameroon, 2014-2018.

A total of 11118 subjects were invited to participate in the study, 305 (2.7%) of them declined our invitation, and 144 (1.3%) were excluded for not completing the questionnaire. Among the 10669 participants eligible, 5055 had a valid prebronchodilator spirometry, and 5021 ultimately provided fully valid data (Figure 1).

The age of our participants ranged from 19 to 96 years, with a median (25^th^-75^th^ percentile) of 43 (30–56) years. Forty percent of them were less than 40 years of age. The sex ratio was 0.8. Nearly 20% of our sample was illiterate, and 12.7% had reached the university level. More than 2/3 of the participants came from an urban setting, and only 20% were ranked as having a high socioeconomic level. The Semi-Bantu ethnic group (54.7%) was the most frequent. Eight hundred and twenty-seven (16.4%) of the subjects admitted they had smoked tobacco, and 81.0% of them had smoked ≤ 20 pack-years (PY). Only 3755 participants, of whom only 20% admitted regular consumption, reported alcohol consumption. Biomass fuel exposure was present in 68.2% of the whole sample and 96.1% of the periurban-rural settings. A history of tuberculosis, pneumonia, asthma, and diabetes mellitus were present in less than 3% of the sample for each. Chronic cough and chronic expectoration had a low frequency. Sixty percent of the enrolled population were overweight or obese. These baseline characteristics are presented in Table 1.

### 3.2. Spirometry and COPD

A total of 5021 participants performed valid spirometry with postbronchodilator measurements. Median prebronchodilator FEV1 and FVC were 2.5 (1.9, 3.1) L and 3.0 (2.3, 3.6) L, respectively. One hundred and forty-five participants met the definition of COPD, giving a prevalence (95% CI) of 2.9% (2.4, 3.3). Data on COPD severity and risk factors are shown in Table 2. When the sample was restricted to participants ≥ 40-year-old, 111/2 977 of them met the definition of COPD, giving a prevalence of 3.7% (3.0, 4.4).

### 3.3. COPD Predictors

In univariate analysis, several factors were associated with COPD. There was a positive correlation with age as both a continuous variable and a categorical one, with an increasing crude OR with respect to age category, taking 19-39 years as reference. Men were more likely to have COPD than women (3.4% vs. 2.4%), but the difference was not significant. Compared with illiterate participants, educated ones were more prone to COPD, and cORs were proportional to the level of education. COPD was also associated with a low socioeconomic level, Semi-Bantu, and other ethnic groups compared to Bantu, tobacco smoking, biomass fuel exposure, history of tuberculosis, history of asthma, chronic bronchitis, and dyspnea. Obese people were less likely to present COPD compared with people of normal weight. Data from univariate analysis are detailed in Table 3.

Among the 16 variables eligible for multivariate step-down analysis, 3 (alcohol consumption, chronic bronchitis, and chronic expectoration) were withdrawn due to excessive missingness. Thus, 13 variables entered the process of multiple logistic regression analysis. Smoking was kept only in its dichotomic form. At the end of this process, seven factors emerged as independent predictors of COPD: educational level, with the highest aOR corresponding to the university level; living in a semiurban or rural locality; tobacco smoking; biomass fuel exposure; experience of dyspnea; history of tuberculosis; and history of asthma. Obesity appeared to be protective against COPD, after adjustment for other confounders. The multivariate analysis is detailed in Table 4.

## 4. Discussion

Our study revealed a 2.9% prevalence of COPD-LLN among Cameroonians aged 19 years and older. Independent predictors of this condition were advanced educational level, suburban or rural residency, tobacco smoking, biomass fuel exposure, the presence of dyspnea, history of tuberculosis, and asthma, while being obese appeared to be a protective factor. Factors associated with COPD in univariate analysis included age, male gender, low socioeconomic level, non-Bantu ethnic groups, and chronic bronchitis.

Regarding epidemiological and clinical features, we can group these factors in 3 main COPD-related categories: (1) risk factors (age, tobacco smoking, biomass fuel exposure, history of tuberculosis, and asthma), (2) clinical manifestations (dyspnea and chronic bronchitis), and (3) confounders (gender, level of education, residency, socioeconomic level, ethnic group, alcohol consumption, and weight category).

In our study, we chose 19-year-olds as the threshold rather than 40-year-olds. This was driven by the knowledge of local features of COPD, which may involve a younger population, compared with western settings. LMIC-specific risk factors such as biomass fuel exposure or a history of tuberculosis, combined with a lower prevalence of asthma, are thought to allow this higher involvement of young subjects in COPD.

Our COPD prevalence was relatively low, even when the sample was limited to participants ≥ 40 years old (3.7%, 95% CI = 3.0%-4.4%). However, this prevalence is consistent with the recent findings on FEV1/FEV LLN-based COPD in SSA, as shown in Table 5. Of the 8 studies that used FEV1/FVC less than the LLN to define COPD (15, 24 28–30, 32–34), 5 showed a prevalence < 10% and half of them were ≤5% (Table 5). Studies that used the fixed FEV1/FVC ratio to define COPD were more likely to show a higher COPD prevalence [7, 25, 26, 33, 39, 40]. This phenomenon is thought to reveal an overestimation of COPD when using a fixed ratio, due to its physiological decline with age. In fact, FEV1/FVC values ranging between 0.65 and 0.7 are common in the elderly and are frequently higher than the related LLN. However, no matter the definition used to define COPD, data in Table 5 show that study populations aged ≥ 30-40 years [15, 25, 26, 28, 33, 39, 41] had higher COPD prevalence (range 9.3%–22.8%) than those aged ≥ 18-20 years (range 2.0%–8.03%) [11, 24, 29, 31, 32, 34]. This supports the identification of advancing age as a significant risk factor for COPD, wherever the study occurs.

Nevertheless, age was the sole known risk factor that did not remain significantly associated with COPD after multivariate analysis in our study, even though it has been widely documented in recent SSA-related literature [5, 7, 8, 25, 26, 31, 33, 34, 42]. Our age distribution, involving an overrepresentation of younger people (40.5% < 40 years and 79.8% < 60 years) who were at lower risk of COPD, as well as the great number of confounding factors involved, could partially explain this result. Tobacco smoking was confirmed as a strong predictor of COPD in this study, and the known dose-effect phenomenon has been clearly demonstrated. However, tobacco smoking exposure (16.4%) was quite lower in our sample than exposure to biomass fuel (68.2%), and its aOR was smaller than any of the other 3 risk factors (biomass fuel exposure, history of tuberculosis, and history of asthma). This is consistent with the observation that more people are exposed to biomass fuel than to tobacco smoking worldwide [12, 14] and especially in SSA [26, 28, 43]. This also assumes that in our setting or in LMIC, these alternative etiologies are more powerful than tobacco in causing COPD. Such an assumption is confirmed by recent studies that assessed simultaneously tobacco smoking and at least one of the four other risk factors as predictors of COPD, summarized in Table 6 [8, 15, 19–21, 24–26, 28, 30, 32, 42]. Of the 9 studies that assessed tobacco smoking in LMIC (excluding the Korean study [19] and 2 others derived from the BOLD study [8, 15]), only Woldeamanuel et al.'s one in Ethiopia showed tobacco smoking to be more strongly associated with COPD than the other risk factors [25]. Interestingly, even in the BOLD study, which occurred mainly in high-income countries and included only South Africa as a SSA country (over 14), Hooper et al. found an OR for a history of tuberculosis higher than that for tobacco smoking [15]. When restricted to never smokers, the database analysis found a history of asthma as a significant associated risk factor, while passive smoking was not [8]. Those studies also reveal a history of tuberculosis as the most potently associated risk factor in LMIC.

The association of COPD with its clinical manifestations (dyspnea on exertion and chronic bronchitis) was anticipated and has been described by other authors [28, 42]. Low socioeconomic status has been associated with COPD [5, 7, 23]. This was found in the present study only in the univariate analysis, probably due to the impact of other confounders. The association between COPD and low economic status seems logical as the poorest people are more exposed to potentially risk factors such as tuberculosis and biomass fuel, and even tobacco smoking. Being literate, which is not usually associated with low socioeconomic status, was strongly associated with COPD in our study, in a dose-effect manner. Similar results were shown by Hooper et al. on never smokers and Lamprecht et al., both from the BOLD study [8, 15]. An approach to explain this discrepancy could be that these findings are linked to differences in perceptions, attitudes, and awareness with regard to tobacco smoking (which is the most common risk factor worldwide). Literate people in developed and western settings do not smoke much as they are more aware of tobacco's noxiousness, while in Cameroon, student smoking has been associated with classmates smoking, advancing in age, and attending public education [44, 45]. The higher frequency of biomass smoke exposure in semiurban or rural residences compared to urban ones (1 428/1 486 = 96.1% vs. 2 018/3 569 = 56.5%, *p* < 0.001) could explain the association of the former with COPD.

As reported by Lamprecht et al. in 2011 and Hooper et al. in 2012 from the BOLD study, obese people were less likely to have COPD in our study. This is also consistent with the results found by Pefura-Yone et al. in his facility-based case-control study in 2015, where COPD was associated with a 1 kg/m^2^-decrease in BMI (aOR = 1.17 (1.01-1.35), *p* = 0.036). Smoking is usually accompanied by a decreased propensity to eat; thus, obesity is less likely to occur among smokers and subsequently among COPD carriers. This was verified in our study, where the prevalence of obesity was significantly higher in never smokers than in ever smokers (1 243/4 209 = 29.5% vs. 133/825 = 16.1%, *p* < 0.001). Conversely, obesity is often associated with higher socioeconomic status in our setting, the latter also being protective against COPD. This was also verified in our study, where obesity prevalence according to socioeconomic status ranged from 23.7% (473/1992) in the low class to 32.2% (325/1009) in the high class (*p* < 0.001).

As for any cross-sectional study, this one could not check the causality between identified risk factors and COPD. However, those we found here are well known and have been identified as such in prospective studies [20, 28]. Choosing the LLN threshold to define COPD could make the comparison hazardous between our results and those of studies that used 0.7 ratio, which are more frequent. Limitations of the fixed ratio have been discussed above. Moreover, recent studies on COPD are more likely to use the LLN than the fixed FEV1/FVC ratio since there is an increasing awareness of these limitations. In our study, the use of FEV1/FVC = 0.7 for COPD definition gave a prevalence of 3.4% (2.9, 3.9)%, which does not significantly differ from the one found using the LLN threshold. A similar argument can be made about our threshold age of 19 years rather than 40 years, which has been widely used so far. The latter was historically chosen because of the large predominance of tobacco smoking as an etiology, given the delay required before developing COPD. Another factor for choosing 40-year threshold was the rarity of asthma development after that age, minimizing the risk of confusion. Recent observations and findings on alternative risk factors, such as history of tuberculosis and biomass exposure, should encourage the use of a lower age threshold for COPD definition. Self-reporting of various exposures may have misclassified some participants. However, the consistency of our results with known epidemiology and related literature makes us feel that there has been no significant bias.

To the best of our knowledge, our study is the largest community-based study ever conducted in Africa to estimate the prevalence of COPD and identify its predictors. Moreover, we think that the rigorous randomized selection process of participants and the multicenter nature of the study made our sample more representative of the Cameroonian population. The large sample size allowed simultaneous analysis of multiple factors, leading to the identification of 8 predictors after multivariate analysis.

## 5. Conclusion

The current study confirmed previous findings about the low prevalence of COPD in SSA compared to western settings, as well as new risk factors. It is thereby contributing to improving knowledge on COPD in this subregion. Being aware of these findings may encourage the population to avoid hazardous behaviours, clinicians to actively seek COPD when facing those risk factors, and finally decision-makers to undertake efficient measures against the identified factors.

## Figures and Tables

**Table 1:** Characteristics of the population enrolled in COPD community study, Cameroon, 2014-2018. Categorical data are expressed as counts (%) and continuous data as median (1^st^, 3^rd^ quartiles).

Sections	Variables	Sample size	Values
Sociodemographic data	**Age, continuous (years)**	5 055	43 (30, 56)
	**Age, categorical (years)**	5 055	
	19–39		2 046 (40.5)
	40-59		1 989 (39.3)
	60-79		921 (18.2)
	80+		99 (2.0)
	**Sex**	5 055	
	Female		2 774 (54.9)
	Male		2 281 (45.1)
	**Education**	5 051	
	University		641 (12.7)
	Secondary		1 226 (24.3)
	Primary		2 185 (43.3)
	None		999 (19.8)
	**Residency**	5 055	
	City		3 569 (70.6)
	Suburban or rural		1 486 (29.4)
	**Marital status**	5021	
	Couple		2 356 (46.8)
	Single		2 675 (53.2)
	**Socioeconomic level**	5 022	
	High		1 011 (20.1)
	Intermediate		2 018 (40.2)
	Low		1 993 (39.7)
	**Ethnic group**	5 046	
	Bantu		1 556 (30.8)
	Semi-Bantu		2 761 (54.7)
	Sudanese, Fulani, or mixed		729 (14.4)

Lifestyle and exposures	**Tobacco smoking**	5 038	827 (16.4)
	**Smoking in pack-years**	5 038	
	None		4 211 (83.6)
	≤20		670 (13.3)
	21–39		99 (2.0)
	≥40		58 (1.2)
	**Alcohol consumption**	3 755	
	Abstinent		299 (8.0)
	Former		2463 (65.7)
	Occasional		219 (5.8)
	Regular		774 (20.6)
	**Biomass fuel exposure**	5 055	3 446 (68.2)

Past medical history	**History of tuberculosis**	5 053	135 (2.7)
	**History of asthma**	5 050	126 (2.5)
	**History of pneumonia**	5 050	117 (2.3)

Comorbidities	**Heart disease**	5 051	44 (0.9)
	**Hypertension**	5 053	438 (8.7)
	**Diabetes mellitus**	5 052	132 (2.6)

Clinical presentation	**Chronic bronchitis**	2 610	99 (3.8)
	**Chronic expectoration**	1 358	86 (6.3)
	**Dyspnea**	5 055	485 (9.6)
	**Weight category**	5 051	
	Normal		1 921 (38.0)
	Lean		111 (2.2)
	Overweight		1 635 (32.4)
	Obese		1 384 (27.4)

COPD = chronic obstructive pulmonary disease.

**Table 2:** Spirometry data of the population enrolled in COPD community study, Cameroon, 2014-2018. Categorical data are expressed as counts (%) and continuous data as median (1^st^, 3^rd^ quartile).

Variables	Sample size	Values
Prebronchodilator FEV1 (liter)	5 021	2.5 (1.9, 3.1)
Prebronchodilator FVC (liter)	5 021	3.0 (2.3, 3.6)
Postbronchodilators FEV1/FVC < LLN (COPD)	5 021	145 (2.9)
COPD severity	145	
Mild		23 (15.9)
Moderate		84 (57.9)
Severe		31 (21.4)
Very severe		7 (4.9)
COPD etiologies	145	
Biomass exposure		126 (86.9)
Smoking		39 (26.9)
History of asthma		16 (11.0)
History of tuberculosis		15 (10.3)

COPD = chronic obstructive pulmonary disease, FEV1 = forced expiratory volume after 1 second, FVC = forced vital capacity, and LLN = lower limit of normal.

**Table 3:** Univariate analysis of factors associated with COPD in the COPD community study, Cameroon, 2014-2018.

Variables	Sample size/counts	COPD *N* (%)	Crude OR (95% CI)	*p* value (Wald)	*p* value (LR)
Age, continuous (years)	5 021	/	**1.03 (1.02, 1.04)**	**<0.001**	**<0.001**
Age, categorical (years)	5 021				**<0.001**
19–39	2 044	**34 (1.7)**	**1**	/	
40-59	1 969	**58 (2.9)**	**1.8 (1.2, 2.7)**	**0.007**	
60-79	912	**42 (4.6)**	**2.8 (1.7, 4.5)**	**<0.001**	
80+	96	**11 (11.5)**	**7.6 (3.7, 15.6)**	**<0.001**	
Sex	5 021				**0.079**
Male	2 268	**78 (3.4)**	**1**		
Female	2 753	**67 (2.4)**	**0.7 (0.5, 1.0)**	**0.078**	
Higher level of education	5 017				**<0.001**
None	998	**8 (0.8)**	**1**		
Primary	2 181	**51 (2.3)**	**2.9 (1.4, 6.3)**	**0,004**	
Secondary	1 215	**44 (3.6)**	**4.6 (2.2, 9.9)**	**<0.001**	
University	623	**41 (6.6)**	**8.7 (4.1, 18.7)**	**<0.001**	
Residency	5 021				**<0.001**
City	3 564	**66 (1.9)**	**1**	/	
Suburban or rural	1 457	**79 (5.4)**	**3.0 (2.2, 4.2)**	**<0.001**	
Marital status	4 997				0.178
Couple	2 655	85 (3.2)	1		
Single	2 342	60 (2.6)	1.3 (0.9, 1.8)	0.178	
Socioeconomic level	4 988				**0.02**
High	1 010	**22 (2.2)**	**1**		
Intermediate	2 009	44 (2.2)	1.0 (0.6, 1.7)	0.983	
Low	1 969	**78 (4.0)**	**1.8 (1.1, 3.0)**	**0.012**	
Ethnic group	5 012				**<0.001**
Bantu	1 556	**24 (1.5)**	**1**		
Semi-Bantu	2 736	**97 (3.5)**	**2.4 (1.5, 3.7)**	**<0.001**	
Sudanese, Fulani, or mixed	720	**24 (3.3)**	**2.2 (1.2, 3.9)**	**0.007**	
Tobacco smoking	5 004				**0.001**
No	4 183	**106 (2.5)**	**1**		
Yes	821	**39 (4.8)**	**1.9 (1.3, 2.8)**	**<0.001**	
Smoking category (pack-years)	5 004				**0.001**
None	4 183	**106 (2.5)**	**1**	/	
<20	666	**26 (3.9)**	**1.6 (1.0, 2.4)**	**0.045**	
21-40	98	**7 (7.1)**	**3.0 (1.3, 6.5)**	**0.007**	
>40	57	**6 (10.5)**	**4.5 (1.9, 10,8)**	**<0.001**	
Alcohol consumption	3 721				**0.08**
Abstinent	297	8 (2.7)	1		
Former	2 441	79 (3.2)	1.2 (0.6, 2.5)	0.615	
Occasional	217	**18 (8.3)**	**3.2 (1.4, 7.7)**	**0.006**	
Regular	766	**28 (3.7)**	**1.4 (0.6, 3.0)**	**0.438**	
Biomass fuel exposure	5 021				**<0.001**
No	1 607	**19 (1.2)**	**1**	/	
Yes	3 414	**126 (3.7)**	**3.2 (2.0, 5.2)**	**<0.001**	
History of tuberculosis	5 019				**<0.001**
No	4 886	**130 (2.7)**	**1**		
Yes	133	**15 (11.3)**	**4.6 (2.5, 8.0)**	**<0.001**	
History of asthma	5 016				**<0.001**
No	4 890	**129 (2.6)**	**1**		
Yes	126	**16 (12.7)**	**5.4 (3.0, 9.1)**	**<0.001**	
History of pneumonia	5 016				0.144
No	4 900	139 (2.8)	1		
Yes	116	6 (5.2)	1.9 (0.7, 4.0)	0.144	
Heart disease	5 017				0.807
No	4 973	144 (2.9)	1		
Yes	44	1 (2.3)	0.8 (0.04, 3.6)	0.807	
Hypertension	5 019				0.259
No	4 583	136 (3.0)	1		
Yes	436	9 (2.1)	0.7 (0.3, 1.2)	0.285	
Diabetes	5 018				0.520
No	4 888	139 (2.8)	1		
Yes	130	5 (3.8)	1.4 (0.5, 3.1)	0.501	
Chronic bronchitis	2 585				**0.001**
No	2 488	**71 (2.9)**	**1**		
Yes	97	**13 (13.4)**	**5.3 (2.7, 9.6)**	**<0.001**	
Chronic expectoration	1 333				0.278
No	1 248	66 (5.3)	1		
Yes	85	7 (8.2)	1.6 (0.6, 3.4)	0.252	
Dyspnea	5 021				**<0.001**
No	4 540	**114 (2.5)**	**1**		
Yes	481	**31 (6.4)**	**2.7 (1.7, 4.0)**	**<0.001**	
Weight category					**<0.001**
Normal	1 908	**73 (3.8)**	**1**		
Thin	107	7 (6.5)	1.8 (0.8, 3.9)	0.167	
Overweight	1 624	48 (3.0)	0.8 (0.5, 1.1)	0.157	
Obese	1 378	**17 (1.2)**	**0.3 (0.2, 0.5)**	**<0.001**	

COPD = chronic obstructive pulmonary disease, OR = odds ratio, CI = confidence interval, and LR = likehood ratio. For variables associated with COPD at threshold 0.10, counts, frequencies, odd ratios and their confident intervals are presented in boldface.

**Table 4:** Multivariate analysis of factors associated with COPD in the COPD community study, Cameroon, 2014-2018. *N* = 4 953.

Variables	Univariate	Initial model	Final model		
	cOR (95% CI)	aOR (95% CI)	aOR (95% CI)	*p* value (Wald)	*p* value (LR)
Age, continuous (years)	1.03 (1.02, 1.04)	1.0 (0.97,1.04)			
Age, categorical (years)					
19–39	1				
40-59	1.8 (1.2, 2.7)	0.9 (0.4, 2.0)			
60-79	2.8 (1.7, 4.5)	0.8 (0.2, 3.1)			
80+	7.6 (3.7, 15.6)	1.3 (0.2, 8.7)			
Sex					
Male	1	1			
Female	0.7 (0.5, 1.0)	0.7 (0.4, 1.0)			
Level of education					**0.001**
None	1	**1**	**1**	/	
Primary	2.9 (1.4, 6.3)	**2.9 (1.3, 6.6)**	**2.7 (1.2, 6.0)**	**0.015**	
Secondary	4.6 (2.2, 9.9)	**3.4 (1.4, 8.1)**	**3.1 (1.3, 7.1)**	**0.009**	
University	8.7 (4.1, 18.7)	**5.6 (2.1, 14.7)**	**4.7 (2.0, 11.1)**	**<0.001**	
Residency					**<0.001**
City	1	**1**	**1**	/	
Semiurban or rural	3.0 (2.2, 4.2)	**1.7 (1.1, 2.7)**	**2.0 (1.4, 3.0)**	**<0.001**	
Socioeconomic level					
High	1	1			
Intermediate	1.0 (0.6, 1.7)	0.7 (0.4, 1.2)			
Low	1.8 (1.1, 3.0)	0.9 (0.5, 1.5)			
Ethnic group					
Bantu	1	1			
Semi-Bantu	2.4 (1.5, 3.7)	1.3 (0.7, 2.2)			
Sudanese, Fulani, or mixed	2.2 (1.2, 3.9)	0.7 (0.4, 1.5)			
Smoking					**0.014**
No	1	**1**	**1**	/	
Yes	1.9 (1.3, 2.8)	**1.4 (0.9, 2.2)**	**1.7 (1.1, 2.5)**	**0.011**	
Biomass smoke exposure					**0.016**
No	1	**1**	**1**	/	
Yes	3.2 (2.0, 5.2)	**2.0 (1.2, 3.6)**	**1.9 (1.1, 3.3)**	**0.021**	
Dyspnea					**<0.001**
No	1	**1**	**1**	/	
Yes	2.7 (1.7, 4.0)	**2.2 (1.4, 3.5)**	**2.2 (1.4, 3.5)**	**<0.001**	
History of tuberculosis					**<0.001**
No	1	**1**	**1**	/	
Yes	4.6 (2.5, 8.0)	**3.6 (1.9, 6.8)**	**3.6 (1.9, 6.7)**	**<0.001**	
History of asthma					**<0.001**
No	1	**1**	**1**	/	
Yes	5.4 (3.0, 9.1)	**6.6 (3.5, 12.2)**	**6.3 (3.4, 11.6)**	**<0.001**	
Weight category					**<0.001**
Normal	1		**1**	/	
Lean	1.8 (0.8, 3.9)	**1.2 (0.5, 3.0)**	**1.1 (0.5, 2.6)**	**0.833**	
Overweight	0.8 (0.5, 1.1)	**0.7 (0.5, 1.1)**	**0.7 (0.5, 1.1)**	**0.162**	
Obese	0.3 (0.2, 0.5)	**0.3 (0.2, 0.5)**	**0.3 (0.2, 0.5)**	**<0.001**	

COPD = chronic obstructive pulmonary disease, c/a OR = crude/adjusted odds ratio, CI = confidence interval, and LR = likehood ratio. For variables associated with COPD at threshold 0.05, counts, frequencies, odd ratios and their confident intervals are presented in boldface.

**Table 5:** COPD prevalence in spirometry-based studies in Sub-Saharan Africa.

Feature	N°	First author, year (reference)	Country	Study design	Population Sample size	Age in years	FEV1/FEV cut-off	Prevalence (%)
COPD-LLN	1a	Akanbi, 2015 [33]	Nigeria	Cross-sectional, hospital-based	HIV infected aged ≥ 30 356	44.5 ± 7.1^∗^	/	22.19
	2	Hooper, 2012 [15]	South Africa	Cross sectional, community-based	Aged >40 yr. 842	NA	/	Male: 22.8 Female: 16.8
	3	Kayondo, 2020 [30]	Uganda	Cross-sectional, facility-based	Rural, HIV- infected adults. 722	40.0	/	6.22
	4	Gemert, 2015 [28]	Uganda	Prospective cohort	Rural, aged > 30. 588	45.0 ± 13.7^∗^	/	16.2
	5	North, 2019 [29]	Uganda	Cross-sectional, community-based	Rural, aged ≥ 18. 565	39.0 ± 17.0^∗^	/	2.0
	6	Pefura-Yone, 2015 [32]	Cameroon	Case control (outcome = HIV infection), facility-based	Patients, aged > 18. 922	42.1 ± 10.6^∗^	/	HIV+: 5.2 HIV-: 5.0
	7a	Pefura-Yone, 2016 [34]	Cameroon	Cross-sectional, community-based	Urban, aged ≥19. 1 287	34.4 ± 12.8^∗^	/	2.4
	8a	Zoller, 2018 [24]	Tanzania	Cross-sectional, primary healthcare facility-based	Patients and visitors, aged ≥ 18. 598	46 (37-57)^*α*^	/	4.0

COPD <0.7	1b	Akanbi, 2015 [33]	Nigeria	Cross-sectional, hospital-based	HIV infected, aged ≥ 30. 356	44.5 ± 7.1^∗^	0.7	15.4
	9	Buist, 2007 [39]	South Africa	Cross-sectional (from BOLD study)	Urban, aged > 40. 896	/	0.7 and FEV1<80%	24.8
	10	Fullerton, 2011 [7]	Malawi	Cross-sectional	Biomass exposed. 372	/	0.7	16.0
	11	Gathuru, 2002 [41]	Nigeria	Cross-sectional	Urban civil servants, aged ≥ 30. 410	47.8 (30-69)^*α*^	0.7	9.3
	12	Gridler-Brown, 2008 [40]	South Africa, Lesotho	Cross-sectional	Urban, former goldminers. 620	49.4 (25.9-61.7)^*β*^	0.7	13.5
	13	Magitta, 2018 [26]	Tanzania	Cross-sectional	Adults aged ≥ 35. 869	51.8 ± 10.6^∗^	0.7	17.5
	14	Meghji, 2016 [31]	Malawi	Cross-sectional, community-based	Adults aged ≥ 18. 749	41.9 ± 15.3	0.7	Male: 4.3 Female: 4.1
	15	Martins, 2009 [11]	Cape Verde	Cross-sectional, primary healthcare facility-based	Out-patients aged > 20. 274	38 (28-50)^*α*^	0.7 and FEV1<80%	8.03
	7b	Pefura-Yone, 2015 [32]	Cameroon	Case control (outcome = HIV infection)	Facility-based, patients aged >18. 922	42.1 ± 10.6^∗^	0.7	HIV+2.2: HIV-: 0.7
	16	Woldeamanuel, 2019 [25]	Ethiopia	Cross sectional, Abeshge District	Adults aged ≥ 30. 734	39.15 ± 9.36^∗^	0.7	17.8
	8b	Zoller, 2018 [24]	Tanzania	Cross-sectional, primary healthcare facility-based	Patients and visitors, aged ≥18. 598	46 (37-57)^*α*^		5.0

^∗^ *Mean* ± *standard* *error*. ^*α*^Median (1^st^–3^rd^ quantiles). ^*β*^Mean (lowest-highest). NA = not available.

**Table 6:** Main results of recent studies on the prediction of COPD in SSA.

N°	Author (reference)	Country, year of publication	Design, population, and sample size	Measure of association	Strength of association				
					Age	Tobacco smoking	History of TB	Biomass fuel exposure	History of asthma
1	Gemert et al. [28]	Uganda 2015	Prospective cohort, Rural >30 yr, 588	aOR	NS	NS	NA	NS	NA
2	Hooper et al. [15]	International^*α*^, 2012	Cross-sectional, community-based, >40 yr, 9 606	oAR	2.14^*β*^	1.34	1.72	NS	NA
3	Idolor et al. [21]	Philippines, 2011	Cross-sectional, >40 yr, 722	aOR	NS	2.86	6.31	3.48	NA
4	Kayondo et al. [30]	Uganda, 2020	Cross-sectional, rural facility-based, HIV-infected adults, 722	aOR	NA	NS	4.92	NA	NA
5	Lamprecht et al. [8]	International^*α*^, 2011	Cross-sectional, community-based, >40 yr, 4 291	aOR	NS	NS^§^	NS	NS	4.12/4.62^*μ*^
6	Lee et al. [19]	Korea, 2011	Cross sectional, >18 yr, 3 687	aOR	1.12^*β*^	2.18	2.64	NA	NA
7	Lee et al. [20]	Taiwan, 2012	Cohort, nationwide, adults, 19 056	HR	1.047	NA	2.054	NA	NA
8	Magitta et al. [26]	Tanzania, 2018	Cross-sectional, ≥35 yr old, 869	aOR	4.02 (41-50)^∗^ 9.35 (51-60)^∗^ 3.18 (>60)^∗^	1.39	5.93	NA^¥^	NA
9	Mbatchou Ngahane et al. [42]	Cameroon, 2015	Cross-sectional, suburban women ≥40 yr old, 300	a*Δ*FEV1 (in ml)	-27^*β*,*β*^	NS	NA	-120	NA
10	Pefura-Yone et al. [32]	Cameroon, 2015	Case control/HIV, facility-based, >18 yr, 922	aOR	NS	NS	4.27	2.5	NA
11	Woldeamanuel et al. [25]	Ethiopia 2019	Cross sectional, Abeshge District, ≥30 yr, 734	aOR	1.91^∗∗^	4.2	NA	2.2	NA
12	Zoller et al. [24]	Tanzania 2018	Cross-sectional, facility-based, 598	*Δ*FEV1% predicted^€^	NA	NS	6.34	NS	NA

TB = tuberculosis; yr = years old; a = adjusted; OR = odds ratio; NS = not significant; NA = not assessed or not mentioned; HR = hazard ratio. ^*α*^Multicentric BOLD study, 14 countries. *^β^*For 10-year change. ^§^Passive smoking. ^*μ*^Men/women. ^¥^99.5% of population was exposed to biomass fuel. ^∗^Age range in years, ^∗∗^(≥50 vs. <50) years. ^*β*,*β*^For 1-year change. ^€^Difference in forced expiratory volume at the first second's percent of predicted, between exposed and nonexposed.

## Data Availability

The dataset used for our analysis are available upon demand to the supervisor of the study. Any request should be done by email to pefurayone@gmail.com.
